# Post-COVID-19 Pulmonary Function Across Extreme Somatic Constitution (Prakriti) Types: A Cross-Sectional Comparative Study of Male Survivors of Severe and Asymptomatic COVID-19

**DOI:** 10.7759/cureus.116989

**Published:** 2026-09-27

**Authors:** Kiran Tawalare, Pranay Bidwaik, Pallavi Turkar, Aparna S Karnad, Pankaj Jogi, Gunjan Bhusari Shrigiriwar, Mahendra Chakravarti, Sakshi Pali

**Affiliations:** 1 Human Physiology, Shri Ayurved College, Nagpur, IND; 2 Human Physiology, Smt. Vimladevi Ayurvedic Medical College and Hospital, Chandrapur, IND; 3 Human Physiology, Dr. Vedprakash Patil Ayurved College, Jalna, IND; 4 Ayurvedic Pharmacology and Materia Medica, Smt. Vimladevi Ayurvedic Medical College and Hospital, Chandrapur, IND

**Keywords:** covid-19, post-covid-19 condition, prakriti, pulmonary function test, somatic constitution, spirometry

## Abstract

Background: Severe coronavirus disease 2019 (COVID-19) can be associated with persistent pulmonary function abnormalities following recovery, with impaired respiratory function reported among survivors. In Ayurveda, somatic constitution (*prakriti*) refers to an individual's constitutional characteristics and has been proposed to be associated with variations in physiological characteristics and responses to disease. However, evidence regarding differences in post-COVID pulmonary function according to somatic constitution remains limited. This study aimed to compare pulmonary function parameters between male survivors of severe and asymptomatic COVID-19 across *vata*, *pitta*, and *kapha* somatic constitution (*prakriti*) types.

Materials and methods: This cross-sectional analytical study included 90 male COVID-19 survivors aged 21-50 years, comprising 45 survivors of severe COVID-19 and 45 asymptomatic survivors. Each group comprised 15 participants with *vata*, 15 with *pitta*, and 15 with *kapha* somatic constitution. Severe COVID-19 was defined by an HRCT chest severity score >15, whereas asymptomatic participants were individuals with previously RT-PCR-confirmed COVID-19 who did not develop clinical symptoms during the infection period. Somatic constitution was assessed using the Maharashtra University of Health Sciences (MUHS) Somatic Constitution (Prakriti) Assessment Proforma. Pulmonary function was evaluated using spirometry, including percent-predicted forced vital capacity (%FVC), percent-predicted forced expiratory volume in one second (%FEV1), and FEV1/FVC ratio. Data were analyzed using the independent-samples t-test, Pearson's chi-square test, one-way analysis of variance, and Bonferroni-adjusted post-hoc comparisons. A p-value <0.05 was considered statistically significant.

Results: Overall, mean %FVC and %FEV1 were significantly lower among severe COVID-19 survivors than asymptomatic survivors (p = 0.0001 for both), whereas the FEV1/FVC ratio did not differ significantly between the groups (p = 0.96). When analyzed by somatic constitution type, mean %FVC was significantly lower among severe survivors than asymptomatic survivors with *vata* (84.47 vs. 93.93; p = 0.0091) and *pitta* (79.33 vs. 94.60; p < 0.001) somatic constitution, but not among those with *kapha* (90.13 vs. 92.87; p = 0.2087). Similarly, mean %FEV1 was significantly lower among severe survivors than asymptomatic survivors with *vata* (84.27 vs. 94.07; p = 0.0027) and *pitta* (78.00 vs. 93.93; p < 0.001) somatic constitution, but not among those with *kapha* (87.47 vs. 88.60; p = 0.7279) somatic constitution. Within the recovered severe group, pulmonary function also differed across somatic constitution types, with lower %FVC and %FEV1 among *pitta* than *kapha *participants.

Conclusion: Severe COVID-19 survivors with *vata* and *pitta* somatic constitution showed significantly reduced %FVC and %FEV1 compared with asymptomatic survivors with the corresponding somatic constitution types, whereas no significant differences were observed among kapha participants. The relatively preserved FEV1/FVC ratio is compatible with a spirometric pattern suggestive of restriction following severe COVID-19, although true restriction cannot be confirmed by spirometry alone. These findings provide preliminary evidence of variation in post-COVID-19 pulmonary function across somatic constitution types and warrant confirmation through larger prospective studies.

## Introduction

Coronavirus disease 2019 (COVID-19), caused by severe acute respiratory syndrome coronavirus 2 (SARS-CoV-2), has clinical presentations ranging from asymptomatic infection to severe respiratory illness and respiratory failure [1]. Variations in disease severity and recovery highlight the importance of understanding individual responses to infection [2]. Ayurveda describes somatic constitution (prakriti) as an individual's inherent constitutional framework associated with physical, physiological, and psychological characteristics, as well as disease susceptibility [3]. The three major *doshas*, that is, *vata*, *pitta*, and *kapha*, have been described in the cited literature using the functional descriptors kinetic, metabolic, and potential, respectively [4]. Previous ayurgenomics studies have reported measurable genomic and phenotypic variations across extreme *prakriti *types, including differences in gene expression, genetic variants, and biological characteristics [5-7]. These findings provide a biological basis for investigating whether constitutional phenotype may be associated with inter-individual differences in physiological responses and pulmonary function. However, a direct causal relationship between *prakriti* and post-COVID-19 pulmonary recovery has not been established and therefore requires clinical investigation.

Severe COVID-19 may result in persistent pulmonary changes following recovery due to inflammatory injury and structural alterations in lung tissue. Spirometry is an objective method for evaluating pulmonary function and assessing respiratory impairment following COVID-19 [8]. Reverse transcription polymerase chain reaction (RT-PCR) is used to confirm SARS-CoV-2 infection, while high-resolution computed tomography (HRCT) severity scoring helps assess pulmonary involvement and disease severity [9,10]. Individuals with RT-PCR-confirmed COVID-19 who do not develop clinical symptoms during infection are classified as asymptomatic cases [11]. Although post-COVID pulmonary outcomes have been extensively studied, evidence regarding variations in pulmonary function across constitutional types remains limited. We hypothesized that pulmonary function would differ between recovered severe and asymptomatic COVID-19 survivors across extreme somatic constitution types. Therefore, this study aimed to compare pulmonary function parameters between male survivors of severe and asymptomatic COVID-19 across extreme somatic constitution types [12].

## Materials and methods

Study design and setting

This cross-sectional analytical study was conducted at Shri Ayurved College, Nagpur, India, and was reported in accordance with the Strengthening the Reporting of Observational Studies in Epidemiology (STROBE) guidelines [13]. The study was approved by the Institutional Ethics Committee of Shri Ayurved College, Nagpur (approval number: SAMN/IEC/MD/06/2023).

Study duration

The study was conducted from 22 November 2023 to 15 June 2025. Participant screening, recruitment, somatic constitution (*prakriti*) assessment, clinical assessment, and spirometric evaluation were completed during this period after obtaining informed consent. Statistical analysis was performed after completion of data collection.

Selection criteria

The study included male COVID-19 survivors aged 21-50 years who fulfilled the predefined eligibility criteria. Participants had previously been diagnosed with COVID-19 by RT-PCR and were categorized into severe and asymptomatic groups based on their clinical history and disease severity. Recovered severe COVID-19 survivors were defined as male individuals aged 21-50 years with previous RT-PCR-confirmed COVID-19 and an HRCT chest severity score >15, whereas asymptomatic participants were defined as male individuals aged 21-50 years with previous RT-PCR-confirmed COVID-19 who did not develop clinical symptoms during the infection period [14,15]. The study population was further restricted to participants who met the predefined criteria for *vata*, *pitta*, or *kapha *somatic constitution (*prakriti*). This selection enabled a comparison of post-COVID-19 pulmonary function between the two groups across constitutional types.

Sample size

The sample size was initially estimated using the standard formula for a single population proportion, based on the prevalence of COVID-19 antibodies reported in a previous survey conducted in Nagpur [16].

$n = \frac{Z^2 \times p \times (1-p)}{d^2}$

where *n* is the required sample size, *Z* is the standard normal deviate at a 95% confidence level (1.96), *p* is the prevalence of COVID-19 antibodies (0.84), and *d* is the absolute precision (0.10). A prevalence of 84% reported in a previous survey conducted in Nagpur was used for sample-size estimation [16].

Accordingly,

$n = \frac{(1.96)^2 \times 0.84 \times (1-0.84)}{(0.10)^2}$ $n = \frac{3.8416 \times 0.84 \times 0.16}{0.01}$ *n = 42.20* (rounded to 45)

The calculated sample size was approximately 42 and was rounded to 45 participants for group-wise allocation. A total of 343 male individuals who survived COVID-19 were screened for eligibility. Because the primary objective was to compare pulmonary function between recovered severe and asymptomatic COVID-19 survivors, equal numbers were included in the two groups, resulting in a final sample of 90 participants (45 severe and 45 asymptomatic). Quota sampling was used until the required sample size was reached, with 15 participants with *vata*, 15 with *pitta*, and 15 with *kapha* somatic constitution (*prakriti*) in each group.

Data collection and assessment

Data were collected using a structured case record form (CRF). Demographic characteristics, relevant clinical history, previous COVID-19 status, RT-PCR and HRCT findings, HRCT severity score, and COVID-19-related symptoms were recorded. Somatic constitution (*prakriti*) was assessed using the Somatic Constitution (Prakriti) Assessment Proforma incorporated in the Maharashtra University of Health Sciences (MUHS), Nashik, Human Physiology (Kriya Sharir) Practical Record Book under Circular No. 02/2025 [17]. The proforma was used in its prescribed format and was not considered a formally validated assessment scale. It included assessment of physical and constitutional characteristics across *vata*, *pitta*, and *kapha* domains. The proforma contains 35 characteristic features for each domain, which are assessed and scored independently. For the present study, an extreme somatic constitution was operationally defined as a score of ≥70% (at least 25 of 35 characteristics) for a single constitutional type. Participants were therefore classified into one of the three extreme somatic constitution groups based on the predominance of a single constitutional type; scores of two or more constitutional types were not combined for this classification.

Pulmonary function was assessed using spirometry. Spirometry was performed using a Recording Spirometer (RMS India Recorders and Medicare System Private Limited), and the measurements were recorded using RMS Helios 401 software. The pulmonary function parameters assessed included forced vital capacity (FVC), forced expiratory volume in one second (FEV1), and the FEV1/FVC ratio. For each participant, the predicted and obtained values for FVC and FEV1 were recorded, and the corresponding percentage-predicted values (%FVC and %FEV1) were calculated. The percentage of predicted pulmonary function was calculated using the following formula:

$\text{Percentage of predicted value (\%)} = \frac{\text{Obtained value}}{\text{Predicted value}} \times 100$

Accordingly,

$\%FVC = \frac{\text{Obtained FVC}}{\text{Predicted FVC}} \times 100$

$\%FEV_1 = \frac{\text{Obtained FEV}_1}{\text{Predicted FEV}_1} \times 100$

The predicted values were based on individual characteristics including age, height, weight, and sex, while the obtained values represented the measurements recorded during spirometry. The FEV1/FVC ratio was assessed as the proportion of forced vital capacity exhaled during the first second of forced expiration and was used along with %FVC and %FEV1 for pulmonary function comparison.

Study outcomes

Primary Outcome

The primary outcome was to compare pulmonary function parameters (%FVC, %FEV1, and FEV1/FVC) between recovered severe and asymptomatic male COVID-19 survivors across the predefined extreme somatic constitution types.

Secondary Outcomes

The secondary outcomes were the distribution of the three extreme somatic constitution (*prakriti*) types, that is, *vata*, *pitta*, and *kapha*, among recovered severe and asymptomatic COVID-19 survivors; comparison of %FVC, %FEV1, and FEV1/FVC between recovered severe and asymptomatic survivors according to somatic constitution type; and comparison of pulmonary function parameters among participants with *vata*, *pitta*, and *kapha* somatic constitution within the recovered severe group.

Statistical analysis

All collected data were entered into Microsoft Excel (Microsoft Corp., Redmond, WA, USA), cleaned, and analyzed using STATA statistical software version 14.0 (Stata Corp., College Station, TX, USA). Descriptive statistics were used to summarize the study variables. Categorical variables were presented as frequencies and percentages and compared using Pearson's chi-square test. Continuous variables were expressed as mean ± standard deviation (SD) and compared between the recovered severe and asymptomatic groups using the independent-samples t-test. One-way analysis of variance (ANOVA) was used to compare mean pulmonary function parameters among *vata*, *pitta*, and *kapha* somatic constitution (*prakriti*) types within the recovered severe group. Bonferroni-adjusted post-hoc comparisons were performed following statistically significant ANOVA results. The pulmonary function parameters analyzed included %FVC, %FEV1, and FEV1/FVC. A two-tailed p-value < 0.05 was considered statistically significant.

## Results

A total of 90 male COVID-19 survivors aged 21-50 years were included in the study, comprising 45 recovered severe COVID-19 survivors and 45 asymptomatic COVID-19 survivors. Each group consisted of 15 participants with *vata*, 15 with *pitta*, and 15 with *kapha* somatic constitution (*prakriti*). Baseline demographic and anthropometric characteristics are presented in Table 1. The two groups were comparable with respect to age, height, and weight, with no statistically significant differences observed between the groups.

**Table 1:** Comparison of demographic and anthropometric characteristics between recovered severe and asymptomatic COVID-19 survivors Values are presented as mean ± standard deviation (SD). Independent-samples Student's t-test was used for comparison between the recovered severe and asymptomatic groups. A two-tailed p-value < 0.05 was considered statistically significant.

Variable	Asymptomatic group (n = 45), mean ± SD	Recovered severe group (n = 45), mean ± SD	Test statistic	p-value
Age (years)	33.76 ± 7.98	36.67 ± 9.17	t = -1.61	0.1117
Height (cm)	166.93 ± 9.73	166.31 ± 9.20	t = 0.31	0.7560
Weight (kg)	70.24 ± 9.73	69.20 ± 9.20	t = 0.52	0.7237

Mean %FVC and %FEV1 were significantly lower among recovered severe COVID-19 survivors than among asymptomatic COVID-19 survivors (84.64 vs. 93.80 and 83.24 vs. 92.20, respectively; p < 0.001 for both). In contrast, no statistically significant difference was observed in the FEV1/FVC ratio between recovered severe and asymptomatic COVID-19 survivors (98.27 vs. 98.21; p = 0.960) (Table 2).

**Table 2:** Comparison of pulmonary function parameters between recovered severe and asymptomatic COVID-19 survivors Values are presented as mean ± standard deviation (SD). Independent-samples Student's t-test was used to compare the recovered severe and asymptomatic groups. A two-tailed p-value <0.05 was considered statistically significant. FVC: forced vital capacity, FEV1: forced expiratory volume in one second, FEV1/FVC: ratio of forced expiratory volume in one second to forced vital capacity.

Pulmonary function parameter	Asymptomatic group (n = 45), mean ± SD	Recovered severe group (n = 45), mean ± SD	Mean difference	Test statistic	p-value
%FVC	93.80 ± 5.44	84.64 ± 9.91	9.16	t = 5.44	<0.001
%FEV1	92.20 ± 6.02	83.24 ± 10.00	8.96	t = 5.15	<0.001
FEV1/FVC (%)	98.21 ± 4.78	98.27 ± 6.04	0.06	t = −0.05	0.960

According to somatic constitution type, mean %FVC was significantly lower among recovered severe COVID-19 survivors than among asymptomatic COVID-19 survivors with *vata* (84.47 ± 11.39 vs. 93.93 ± 6.42; p = 0.0091) and *pitta* (79.33 ± 8.80 vs. 94.60 ± 4.64; p < 0.001) somatic constitution. No statistically significant difference was observed among participants with *kapha *somatic constitution (90.13 ± 6.25 vs. 92.87 ± 5.34; p = 0.2087). Similarly, mean %FEV1 was significantly lower among recovered severe COVID-19 survivors than among asymptomatic COVID-19 survivors with *vata *(84.27 ± 9.08 vs. 94.07 ± 7.13; p = 0.0027) and *pitta *(78.00 ± 7.66 vs. 93.93 ± 3.15; p < 0.001) somatic constitution, whereas no statistically significant difference was observed among participants with *kapha *somatic constitution (87.47 ± 11.11 vs. 88.60 ± 5.70; p = 0.7279). The FEV1/FVC ratio did not differ significantly between recovered severe and asymptomatic COVID-19 survivors with *vata *(100.00 ± 5.33 vs. 100.03 ± 3.59; p = 0.9873), *pitta* (98.20 ± 2.83 vs. 99.53 ± 3.46; p = 0.2581), or *kapha *(96.60 ± 8.48 vs. 95.07 ± 5.55; p = 0.5627) somatic constitution (Table 3).

**Table 3:** Comparison of pulmonary function parameters between recovered severe and asymptomatic COVID-19 survivors according to somatic constitution type Values are presented as mean ± standard deviation (SD). Independent-samples Student's t-test was used to compare pulmonary function parameters between the recovered severe and asymptomatic groups within each somatic constitution (prakriti) category. A two-tailed p-value <0.05 was considered statistically significant. FVC: forced vital capacity, FEV1: forced expiratory volume in one second, FEV1/FVC: ratio of forced expiratory volume in one second to forced vital capacity.

Pulmonary function parameter	Prakriti	Asymptomatic group (n = 15), mean ± SD	Recovered severe group (n = 15), mean ± SD	Mean difference	Test statistic	p-value
%FVC	Vata	93.93 ± 6.42	84.47 ± 11.39	9.47	t = 2.58	0.0091
	Pitta	94.60 ± 4.64	79.33 ± 8.80	15.27	t = 4.52	<0.001
	Kapha	92.87 ± 5.34	90.13 ± 6.25	2.73	t = 1.28	0.2087
%FEV1	Vata	94.07 ± 7.13	84.27 ± 9.08	9.80	t = 2.93	0.0027
	Pitta	93.93 ± 3.15	78.00 ± 7.66	15.93	t = 6.61	<0.001
	Kapha	88.60 ± 5.70	87.47 ± 11.11	1.13	t = 0.33	0.7279
FEV1/FVC (%)	Vata	100.03 ± 3.59	100.00 ± 5.33	0.03	t = 0.02	0.9873
	Pitta	99.53 ± 3.46	98.20 ± 2.83	1.33	t = 1.08	0.2581
	Kapha	95.07 ± 5.55	96.60 ± 8.48	1.53	t = −0.59	0.5627

Among the recovered severe group, ANOVA demonstrated a statistically significant difference among the *vata*, *pitta*, and *kapha* somatic constitution (*prakriti*) groups for %FVC (F = 5.33, p = 0.0086) and %FEV1 (F = 3.95, p = 0.0270), whereas the difference in FEV1/FVC ratio was not statistically significant (F = 1.20, p = 0.3111). Bonferroni-adjusted post-hoc comparisons demonstrated a statistically significant difference between *pitta* and *kapha* for both %FVC (p = 0.002) and %FEV1 (p = 0.034), whereas the other pairwise comparisons were not statistically significant (Table 4).

**Table 4:** Comparison of pulmonary function parameters among participants with vata, pitta, and kapha somatic constitution (prakriti) in recovered severe COVID-19 survivors Values are presented as mean ± standard deviation (SD). One-way analysis of variance (ANOVA) was used to compare pulmonary function parameters among the *vata*, *pitta*, and *kapha* somatic constitution (*prakriti*) groups in the recovered severe group. Bonferroni-adjusted post-hoc comparisons were performed when the ANOVA was statistically significant. A two-tailed p-value <0.05 was considered statistically significant. FVC: forced vital capacity, FEV1: forced expiratory volume in one second, FEV1/FVC: ratio of forced expiratory volume in one second to forced vital capacity.

Pulmonary function parameter	Vata, mean ± SD	Pitta, mean ± SD	Kapha, mean ± SD	Test statistic	p-value
%FVC	84.47 ± 11.39	79.33 ± 8.80	90.13 ± 6.25	F = 5.33	0.0086
%FEV1	84.27 ± 9.08	78.00 ± 7.66	87.47 ± 11.11	F = 3.95	0.0270
FEV1/FVC (%)	100.00 ± 5.33	98.20 ± 2.83	96.60 ± 8.48	F = 1.20	0.3111

## Discussion

The present study demonstrated significantly lower %FVC and %FEV1 among recovered severe COVID-19 survivors compared with asymptomatic COVID-19 survivors, whereas the FEV1/FVC ratio did not differ significantly between the groups. These findings indicate reduced spirometric pulmonary function among individuals who had recovered from severe COVID-19, particularly in %FVC and %FEV1, while the relatively preserved FEV1/FVC ratio did not suggest a predominant obstructive pattern.

Spirometric parameters such as %FVC, %FEV1, and FEV1/FVC are commonly used to assess pulmonary function and ventilatory impairment. Values of %FVC and %FEV1 ≥80% of predicted and an FEV1/FVC ratio ≥70% are generally considered within normal limits, although interpretation should account for individual characteristics and appropriate reference equations [18]. Percent-predicted values facilitate comparison of pulmonary function by accounting for individual characteristics such as age, sex, height, and ethnicity [19,20]. The present study included males aged 21-50 years to reduce potential variation related to age, as age-related anatomical and physiological changes may influence respiratory function [21]. Additionally, only male participants were included to reduce sex-related variation in spirometric parameters [22,23].

The reduction in %FVC and %FEV1 observed among recovered severe COVID-19 survivors was particularly evident among participants with vata and pitta somatic constitution (prakriti). In contrast, no statistically significant differences were observed between recovered severe and asymptomatic COVID-19 survivors with *kapha* somatic constitution (prakriti). Previous studies have similarly reported reductions in %FVC and %FEV1 following severe COVID-19, suggesting that pulmonary-function abnormalities may persist after recovery [24,25]. These findings may reflect residual pulmonary involvement following severe SARS-CoV-2 infection, although other unmeasured factors cannot be excluded.

In the secondary analysis, pulmonary function parameters were compared among participants with *vata*, *pitta*, and *kapha* somatic constitution (prakriti) within the recovered severe COVID-19 group. Significant differences were observed for %FVC and %FEV1, with the lowest mean values among participants with pitta and the highest among those with *kapha* somatic constitution. Bonferroni-adjusted post-hoc comparisons demonstrated statistically significant differences between *pitta* and *kapha* for both %FVC (p = 0.002) and %FEV1 (p = 0.034), whereas the other pairwise comparisons were not statistically significant. These findings indicate variation in pulmonary function across somatic constitution types within the recovered severe group, but do not establish a causal or effect-modifying relationship. Previous research from Eastern Maharashtra has also evaluated somatic constitution as an individual constitutional characteristic in relation to health-related outcomes, supporting the relevance of somatic constitution assessment in regional populations [26]. Previous studies have reported differences in biological characteristics among somatic constitution types, including inflammatory and regulatory pathways [27]. According to Ayurvedic principles, *agni* is considered fundamental to digestion, absorption, assimilation, and metabolism, with digestive power representing different levels of digestive and metabolic transformation [28]. However, these interpretations remain exploratory and require confirmation in appropriately designed studies.

Previous studies have reported that post-COVID-19 pulmonary-function abnormalities may present with reductions in %FVC and %FEV1 with a relatively preserved FEV1/FVC ratio, consistent with a spirometric pattern suggestive of restriction rather than predominant airway obstruction [29]. A similar pattern was observed in the present study, particularly among participants with *vata* and *pitta* somatic constitution in the recovered severe group. The reduction in %FVC and %FEV1 together with the relatively preserved FEV1/FVC ratio was compatible with this pattern. Such changes may be compatible with residual pulmonary involvement following severe infection [30]. However, because total lung capacity was not directly measured, true restriction cannot be confirmed by spirometry alone.

The findings should be interpreted in the context of the study design. The cross-sectional nature of the study prevents determination of whether the observed differences reflect post-COVID-19 changes associated with previous disease severity, time since COVID-19 recovery, pre-existing differences in pulmonary function, somatic constitution-related variation, or other unmeasured factors. The relatively small sample size and selection of participants with extreme somatic constitution types may also limit the generalizability of the findings. Nevertheless, the study provides preliminary evidence of differences in pulmonary function across somatic constitution types among recovered severe COVID-19 survivors and supports further investigation using larger, prospective, and multicentric studies.

Strengths and limitations

The present study has several strengths. Pulmonary function was objectively assessed using spirometry, and recovered severe and asymptomatic COVID-19 survivors were compared within predefined extreme somatic constitution categories. The inclusion of equal numbers of participants with *vata*, *pitta*, and *kapha* somatic constitution (*prakriti*) in each study group enabled constitution-specific comparisons of pulmonary function. The use of the prescribed somatic constitution (prakriti) assessment proforma also provided a structured approach to constitutional assessment.

The study has certain limitations. The relatively small sample size, single-center setting, male-only population, and selection of participants with extreme somatic constitution may limit the generalizability of the findings. The cross-sectional design prevents establishing temporal or causal relationships between COVID-19 severity, somatic constitution, and pulmonary function. In addition, spirometry alone cannot confirm restrictive lung disease because total lung capacity was not measured. Potential confounding factors, including baseline pulmonary function, smoking status, respiratory comorbidities, other post-COVID-19 factors, psychological stress, environmental exposures such as air pollution, and potential variability in spirometric measurement, may also have influenced pulmonary outcomes. Larger multicentric prospective studies incorporating comprehensive pulmonary-function testing and relevant clinical and biological variables are warranted.

## Conclusions

The present study demonstrated significantly lower %FVC and %FEV1 among recovered severe COVID-19 survivors compared with asymptomatic COVID-19 survivors, particularly among participants with *vata *and *pitta *somatic constitution (*prakriti*), whereas no statistically significant differences were observed among participants with *kapha* somatic constitution. Within the recovered severe group, pulmonary function also differed significantly across somatic constitution types, with participants with *pitta* somatic constitution showing lower %FVC and %FEV1 than those with *kapha* somatic constitution. These findings provide preliminary evidence of variation in post-COVID-19 pulmonary function across somatic constitution types and support further investigation in larger prospective studies.
